# HIV screening in the dental setting in New York State

**DOI:** 10.1371/journal.pone.0231638

**Published:** 2020-04-16

**Authors:** Rakkoo Chung, Shu-Yin John Leung, Stephen N. Abel, Michael N. Hatton, Yanfang Ren, Jeffrey Seiver, Carol Sloane, Howard Lavigne, Travis O’Donnell, Laura O’Shea

**Affiliations:** 1 New York State Department of Health, AIDS Institute, Albany, New York, United States of America; 2 School of Dental Medicine, University at Buffalo, Buffalo, New York, United States of America; 3 Eastman Institute for Oral Health, University of Rochester, Rochester, New York, United States of America; 4 School of Dental Medicine, Stony Brook University, Stony Brook, New York, United States of America; 5 Northeast/Caribbean AIDS Education and Training Center, Syracuse, New York, United States of America; Chinese Academy of Medical Sciences and Peking Union Medical College, CHINA

## Abstract

While primary care providers in New York State (NYS) are mandated to offer all patients a HIV test, still many NYS residents miss the HIV screening opportunity. To fill the gap, and as the CDC recommends, this study aimed to examine the feasibility of implementing HIV screening in dental setting, identify patient characteristics associated with acceptance of HIV rapid testing, and discuss best practices of HIV screening in dental setting. New York State Department of Health (NYSDOH) collaborated with the Northeast/Caribbean AIDS Education and Training Center (NECA AETC) and three dental schools in New York State to offer free HIV screening tests as a component of routine dental care between February 2016 and March 2018. Ten clinics in upstate New York and Long Island participated in the study. HIV screening was performed using the OraQuick™ In-Home HIV Test. 14,887 dental patients were offered HIV screening tests; 9,057 (60.8%) were screened; and one patient (0.011%) was confirmed HIV positive and linked to medical care. Of all dental patients, 33% had never been screened for HIV; and 56% had not had a primary care visit or had not been offered an HIV screening test by primary care providers in the previous 12 months. Multi-level generalized linear modeling analysis indicated that test acceptance was significantly associated with patient’s age, race/ethnicity, gender, country of origin, primary payer (or insurance), past primary care visits, past HIV testing experiences, and the poverty level of patient’s community. HIV screening is well accepted by dental patients and can be effectively integrated into routine dental care. HIV screening in the dental setting can be a good option for first-time testers, those who have not seen a primary care provider in the last 12 months, and those who have not been offered HIV screening at their last primary care visit.

## Introduction

According to the Centers for Disease Control and Prevention (CDC), about 1.1 million persons aged 13 and older were living with HIV infection in the United States by the end of 2015, including an estimated 162,500 (15%) persons whose infection had not been diagnosed.[1] In New York State (NYS), it was estimated that about 122,600 persons were living with HIV, and that about 11,100 of them were unaware of their HIV status.[2] The CDC recommends routine HIV screening in all health care settings to diagnose asymptomatic individuals.[3] Since 2010, NYS HIV Testing Law mandates offering HIV screening tests to patients ages 13–64 who receive hospital or primary care. In 2016, this requirement was expanded to offer HIV screening to individuals over the age of 64.[4] One of the primary goals of the NYS Blueprint to End the AIDS Epidemic[5] is to identify persons living with undiagnosed HIV infection, link them to medical care, and help prevent them from unknowingly transmitting HIV. The NYS Blueprint also recommends routine HIV testing be expanded to additional settings, including dental offices.

Several studies[6–10] have emphasized the potential benefits of routine HIV screening in the dental care setting, especially with the availability of rapid HIV test using oral fluid. Dental care providers (as well as primary care providers) have recognized the importance and the benefits of performing chairside screening for HIV.[8,11–19] In addition, HIV screening in the dental environment provides an opportunity to those who are not screened for HIV during primary care visits. The 2016 Behavioral Risk Factor Surveillance System (BRFSS) data show that approximately two-thirds of individuals over the age of 18 in the United States or in NYS visit a dental office each year, and that those who have never been tested for HIV also show similar rates of dental care utilization in the previous 12 months. Based on 2005 National Health Interview Survey (NHIS) data, 3.6 million Americans were at significant HIV risk yet had never been tested for HIV; and three quarters of them had seen a dentist within the past two years.[20] Surveys have shown that patients have positive attitudes towards rapid HIV screening in dental setting;[21] Over 70% of dental patients report that they would accept an HIV screening test if offered during their dental apointment.[22–25]

The above data suggest that the dental setting is a viable venue in which to implement rapid HIV screening. However, only a few studies have demonstrated implementation of rapid HIV screening in the dental setting in the United States. These studies have shown that patient acceptance varied according to the person offering the screening test and how the offer is presented to the patient. It is suggested that patients are more likely to accept HIV screening if the test is offered by clinical staff members. In a study by Suarez-Durall et al (2019),[26] patients waiting for a dental hygiene appointment were routinely offered a free rapid HIV screening test by trained clinical staff in urban downtown Los Angeles from 2013 to 2016. 319 patients (out of 811) accepted the offer; and one was confirmed HIV positive. In a study by Bradley et al (2018),[27] dentists and hygienists administered rapid oral HIV screening as part of routine care at two dental clinics in South Florida in 2015. A large majority of patients (507 out of 600 patients) accepted HIV screening, but none tested reactive. In a study by Blackstock et al (2010),[28] a full-time trained counselor used whole-blood finger-stick rapid HIV testing to screen dental patients in community clinics in New York City in 2008–2009. Nearly a half of the patients (3,565 out of 7,814) accepted the HIV testing, among whom 19 asymptomatic patients were confirmed to be HIV positive. Nassry et al (2012)[29] offered rapid oral HIV screening at a dental school clinic in New York City. Only 21 out of 256 patients accepted HIV screening when offered by administrative staff. In contrast, 30 out of 34 patients accepted screening when it was offered by a faculty member or a student. No patient showed a reactive test result. In a pilot study by Leung et al (2016),[30] dentists, hygienists, and assistants offered rapid oral HIV screening at dental school clinics in NYS in 2012–2014. Over half of the patients (3,982 out of 7,869) accepted HIV screening, among whom 5 asymptomatic patients were confirmed HIV positive and linked to medical care. Based on a multivariate logistic regression analysis of the data from their large sample, Leung et al (2016)[30] found that recent primary care visits, the lack of prior HIV screening experience (i.e., never been tested for HIV), as well as gender and race/ethnicity, were significant determinants of test acceptance among dental patients.

Considering the discussion in the literature and the data from previously cited studies,[14–15,18,21,23–24] the NYSDOH collaborated with the Northeast/Caribbean AIDS Education and Training Center (NECA AETC) and three NYS dental schools to implement an HIV screening demonstration study at 10 dental clinics. The study was conducted over a two-year period and collected data from a large sample of dental patients. We hypothesized that a patient is more likely to accept HIV screening if the patient (1) had not recently seen a primary care provider, (2) had not been screened for HIV at the recent primary care visit, (3) had never been tested for HIV, (4) had not been screened for HIV recently, and/or (5) resided in an economically disadvantaged community, controlling for the patient’s age, race/ethnicity, gender, country of origin, and dental payer coverage.

## Data and methods

This research was approved by the University at Buffalo Institutional Review Board (MODCR00000542), by the University of Rochester Office for Human Subject Protection (RSRB00060676), and by the Stony Brook University Institutional Review Board (2016-3466-F). Between February 2016 to March 2018, dentists, hygienists, assistants and non-clinical staff offered rapid oral HIV screening tests (OraQuick™ ADVANCE® Rapid HIV-1/2 Antibody Test) free of charge to patients who visited dental clinics associated with the University at Buffalo School of Dental Medicine, University of Rochester Eastman Institute for Oral Health, and School of Dental Medicine at Stony Brook University, and community dental clinics and private practices including Baker Victory Dental Center, Eastman Dental Downtown, Eastman Dental at School #17, the Erie County Health Mall, Seneca Nation Dental Health Services, the Western New York Dental Group, Shinnecock Indian Health, as well as the mobile dental van of Stony Brook University.

The NYSDOH developed the study protocol and the data collection tools, coordinated data submission from the testing sites, managed the centralized database, analyzed the data, and provided dental clinics with funding to purchase the test kits. The NECA AETC provided project coordination, staff training and technical support throughout the project period, and supplied promotional and patient educational materials. The dental school staff conducted HIV screening and collected data at their sites and oversaw data collection by the community dental clinics and private practices.

**Fig 1:**
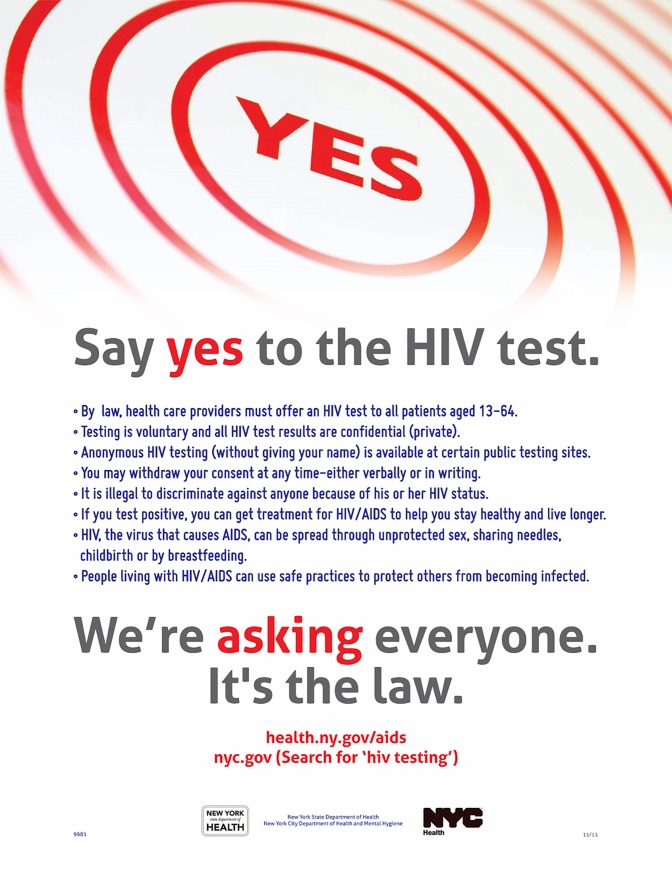
One of the promotional materials displayed in dental clinic waiting areas.

Trained personnel offered an HIV screening test to *all* patients (ages 18 or older) who presented for dental services at the study sites during the 2-year study period. A standard script was used by the dental providers in order to present HIV screening in a calibrated manner across the testing sites. All eligible patients were asked, “Would you like to have an HIV screening test today?” If a patient asked why they were offered an HIV test, testers would respond, “We are offering a free HIV test to everyone.” Also, one of the posters (see Fig 1) displayed at dental offices said, “We’re *asking* everyone. It’s the law.” The screening tests were offered at the beginning of the appointment; and the results were usually ready by the end of the appointment.

If a patient declined the screening offer, the reason for refusal was recorded. If a patient accepted the offer, HIV screening was administered at no cost to the patient. Patients with reactive results were referred for confirmatory testing; they were then linked to medical care if confirmed positive. In addition, we collected data regarding patient demographics, primary care visits in the past year, and previous HIV testing experience for all patients, and any oral signs of HIV infection for those with reactive results.

Data coordinators at each of the dental schools assigned unique numbers to their patients. These numbers were used in the de-identified data and for communication about specific patients with NYSDOH. De-identified data were submitted to NYSDOH using one of the two methods: (1) electronic data files via a secured network, or (2) completed paper forms via certified mail. Data managers at NYSDOH merged the data from all dental clinics, monitored data quality, and provided monthly status reports. NYSDOH staff and dental school representatives held monthly phone conferences to review study data, report implementation barriers and successes, and discuss appropriate solutions to any issues. A qualitative tool was also administered near the end of the demonstration project to solicit input from clinical and non-clinical staff on their evaluation of the HIV screening processes and patients’ responses. Nearly all participating providers shared their experiences with every aspect of the project and reported their perceived barriers, facilitators and best practices.

### Statistical analysis and measures

SAS 9.4 PROC GLIMMIX was used to specify multi-level generalized linear dichotomous models of patient’s acceptance of HIV screening. Patient’s residence was approximated using ZIP Code Tabulated Area (ZCTA). ZCTA was included in the models as random effects to control for unknown characteristics that are shared by the patients residing in the same community. Because the data were collected in ten clinics and a mobile van, test sites were included in the models as fixed effects to control for unknown site effects on patient acceptance.[31–33] We included an additional variable to control for the effects of a protocol change at one of the dental offices (Site #03 in the models). At this site, an HIV screening test was incorporated into one of the routine exams several months after the start of the project.

Patients self-identified their race/ethnicity: Hispanic/Latino, black, white, Native American and Alaskan Native (NAAN), Asian, Native Hawaiian and Pacific Islander (NHPI), and other, multi-racial or unknown race/ethnicity. We calculated patient age based on the year of birth and the year of dental visit, resulting in six age groups. Patients were asked to self-identify their gender as woman, man, transgender woman, or transgender man. Patients’ country of birth was dichotomized to (1) born in the United States or Puerto Rico, or (2) foreign-born. Patients’ primary payer was measured as private coverage, Medicaid, Medicare, and no insurance (i.e., paying out-of-pocket). Patients reported if they had seen a primary care provider in the past 12 months, and, for those had, if they had been offered an HIV test at any of the primary care visits. Patients also reported if they had ever been tested for HIV, and for those had, the month and year of their last HIV test.

Based on patient residence ZIP codes, we linked the patient data to the 2012–2016 American Community Survey 5-year aggregate data. We used the ZIP Code Tabulation Areas (ZCTAs) to measure the following characteristics of patients’ communities: (1) A dummy variable indicating if their ZCTA falls in a metropolitan area, (2) population density (i.e., population sizes divided by the areas of ZCTAs), and (3) the percent of households below the 200% of the federal poverty level. We tested different levels of poverty (from 50% to 500%) and found no noticeable difference in the results. Thus, our findings of the impacts of the community poverty level on patient’s acceptance of an HIV screening offer in our statistical analysis are robust.

## Results

### Patient characteristics

Of 14,935 patients who presented for care at the dental clinics during the project period, 49% were non-Hispanic white, 30% were non-Hispanic black, 12% were Hispanic/Latino, 2% were NAAN, and 2% were Asian. Their age ranged from 18 to 96 with a mean of 46.6 and a median of 46. Female patients (8,051) outnumbered male patients (6,849) and the female-to-male ratio was 1.18. Seven patients self-identified as transgender women. Zero self-identified as transgender men. Nearly half of the patients were covered by Medicaid (46%) or Medicare (3%); 26% paid out-of-pocket; and 17% paid through private insurance (see Table 1).

**Table 1:** Dental patient characteristics.

Characteristics		# (%) of patients	Acceptance^a^
	All	14,935 (100)	60.0
Race/Ethnicity	Hispanic/Latino	1,758 (11.8)	62.9
	Non-Hispanic Black	4,523 (30.3)	56.1
	Non-Hispanic White	7,348 (49.2)	62.2
	Non-Hispanic NAAN^*b*^	343 (2.3)	58.9
	Non-Hispanic Asian	311 (2.1)	64.0
	Non-Hispanic NHPI^*c*^	19 (.1)	47.4
	Other/Multi/Unknown	633 (4.2)	51.3
Age	18 to 24	1,618 (10.8)	64.7
	25 to 34	3,310 (22.2)	62.2
	35 to 44	2,068 (13.8)	56.2
	45 to 54	2,471 (16.5)	55.6
	55 to 64	2,677 (17.9)	57.6
	65 or older	2,749 (18.4)	64.1
	Unknown	42 (.3)	36.6
Gender	Woman	8,051 (53.9)	58.9
	Man	6,849 (45.9)	61.4
	Transgender woman	7 (.0)	42.9
	Transgender man	0 (.0)	-
	Unknown	28 (.2)	50.0
Country of Birth	United States^*d*^	12,540 (84.0)	59.3
	Other country	1,737 (11.6)	68.1
	Unknown	658 (4.4)	55.3
Primary Payer	Private Insurance	2,474 (16.6)	65.2
	Medicaid	6,793 (45.5)	54.2
	Medicare	443 (3.0)	75.8
	No Insurance	3,829 (25.6)	64.9
	Other/Unknown	1,396 (9.3)	54.2
Ever Been Tested for HIV	Yes	7,950 (53.2)	63.8
	No	4,961 (33.2)	70.9
	Unknown	2,024 (13.6)	24.7
Had a Primary Care Visit in the Past 12 Months	Yes	10,448 (70.0)	64.8
	No	2,844 (19.0)	71.2
	Unknown	1,643 (11.0)	17.8
Among Those Who Had a PC Visit in the Past 12 Months, Being Offered HIV Screening	Yes	4,194 (40.1)	55.3
	No	5,583 (53.4)	73.1
	Unknown	671 (6.4)	54.5

^a^ Acceptance rate (%) among the dental patients who were offered an HIV test; data from some dental offices in some months were excluded from the calculation of the acceptance rate if some of the patients who declined the HIV test offer were not reported.

^b^ Native American and Alaskan Native.

^c^ Native Hawaiian and Pacific Islander.

^d^ Including Puerto Rico (PR).

### Offer and acceptance of an HIV screening test

**Fig 2:**
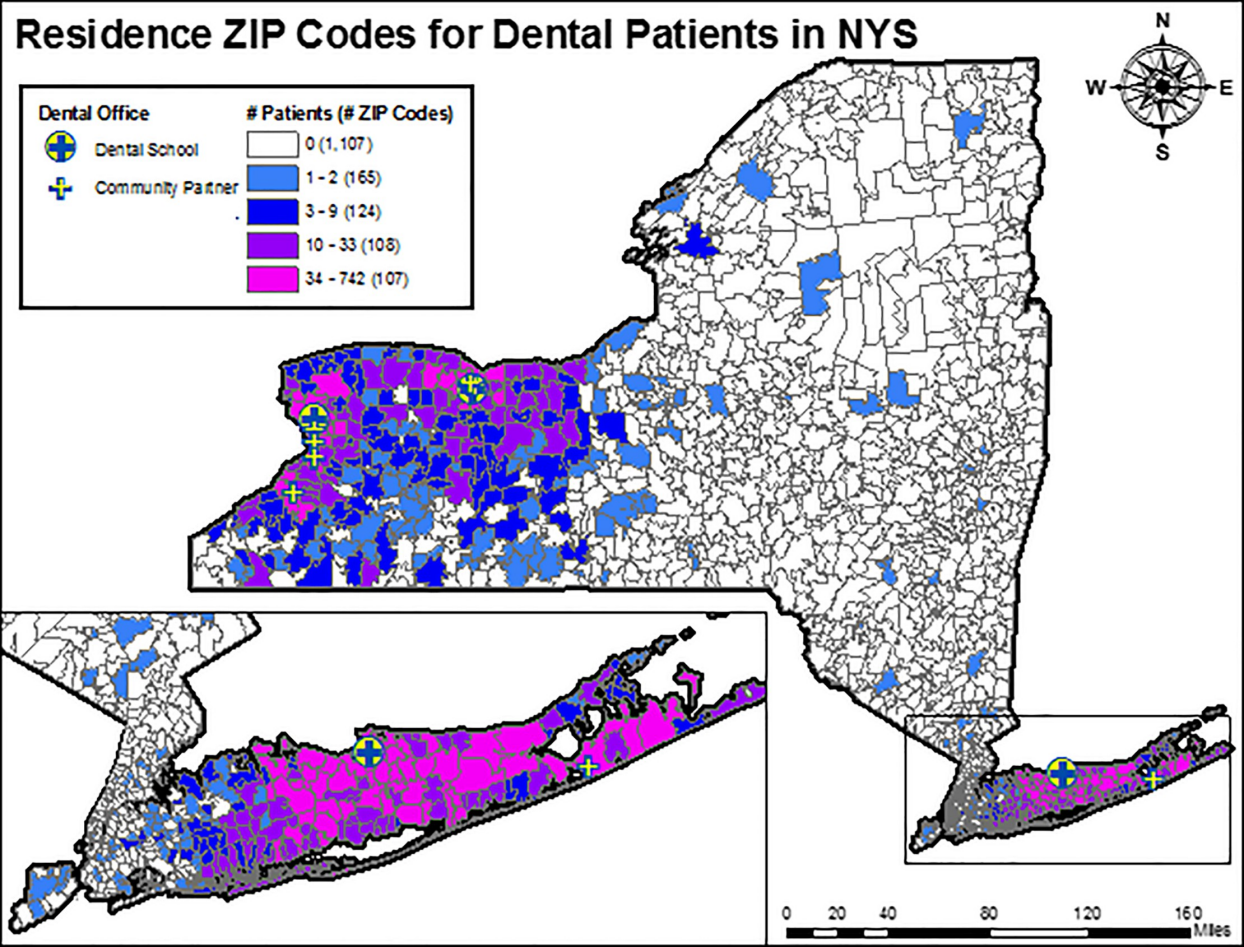
Geographic distribution of dental patients by ZIP Code, 2016–2018.

During the project period, 14,935 patients visited 10 testing sites and one mobile van for dental services; 14,887 (99.7%) were offered an HIV screening test; 9,063 (60.9%) accepted the offer and 9,057 (60.8%) were screened; and one of them was tested reactive, confirmed HIV positive and subsequently linked to medical care. The OraQuick™ In-Home Test made two errors out of the 9,056 tests of uninfected individuals, showing a 99.98% accuracy. Fig 2 shows the geographic distribution of patients using dental services during the study period.

As Table 1 shows, screening acceptance rates varied by patient characteristics, such as race/ethnicity, age, gender, country of birth, and primary payer. Of the 14,935 patients: 4,961 patients (33.2%) had never been tested for HIV when they visited the study sites; 7,950 patients (53.2%) had been tested for HIV at least once in the past; and 2,024 patients (13.6%) did not remember or refused to report this information. Among those who had never been tested for HIV, 71% accepted the free screening offer and were screened for the first time. Furthermore, 8,427 patients (56.4%) did not receive any HIV screening with primary care providers in the previous 12 months, either because 2,844 patients (19.0%) did not have primary care visits or because 5,583 patients (37.4%) were not offered an HIV screening test during their primary care visit in the past 12 months. Among those who had not had an HIV screening opportunity with primary care provider in the past 12 months, 72% were screened for HIV at the project study sites (see Table 1).

The acceptance rate for HIV screening also varied by site. Patient acceptance of the screening test was dependent on how and by whom the screening offer was presented. Screening acceptance rates ranged from 59% to 79% for most clinics except for four sites: two clinics reported having seen fewer than 50 patients during the study period so their acceptance rates were not considered separately; a dental van at community health fairs recorded the highest acceptance rate of 99%; and one site showed the lowest acceptance rate of 13% where the test was offered by non-clinical staff at a reception desk, rather than clinical staff.

At one site (Site #03 in the models), it became evident that the way in which an HIV test offer was presented to patients had a large impact on the acceptance rate. This site’s acceptance rate was 40% for the first months when HIV screening was presented as part of a research study and offered to patients prior to any clinical work. The acceptance rate was around 80% consistently in the rest of the project period when the site made HIV screening a routine part of the overall comprehensive examination for each patient.

Table 2 shows the factors of HIV test acceptance in multivariate analysis. Different sets of variables are introduced in Model 1 through Model 4 to explain patient acceptance. The likelihood ratio test indicates that Model 4, which includes all variables, is the best fitting model for our data.

**Table 2:** Estimates for multi-level generalized linear dichotomous models of HIV test acceptance (n = 13,656).

	Model 1	Model 2	Model 3	Model 4^*a*^
Intercept	.593* (.038)	1.952* (.088)	1.689* (.178)	1.477* (.186)
Age 18–24	-	-	-.027 (.074)	-.027 (.074)
Age 25–34	-	-	reference	reference
Age 35–44	-	-	-.381* (.068)	-.379* (.068)
Age 45–54	-	-	-.371* (.067)	-.369* (.067)
Age 55–64	-	-	-.393* (.069)	-.389* (.069)
Age 65+	-	-	-.403* (.077)	-.397* (.077)
Asian	-	-	.131 (.159)	.100 (.160)
Black	-	-	.357* (.055)	.290* (.058)
Hispanic	-	-	.438* (.074)	.386* (.075)
Multi-Racial	-	-	.768* (.230)	.723* (.230)
NAAN	-	-	.163 (.293)	.108 (.291)
NHPI	-	-	-.360 (.551)	-.352 (.550)
Other	-	-	-.180 (.142)	-.224 (.142)
Unknown race/ethnicity	-	-	-.600 (.334)	-.651 (.334)
White	-	-	reference	reference
Female^*b*^	-	-	-.163* (.041)	-.164* (.041)
Male	-	-	reference	reference
Born in US or PR	-	-	-.205* (.080)	-.204* (.080)
Born elsewhere	-	-	reference	reference
Private coverage	-	-	-.421* (.135)	-.417* (.135)
Medicaid	-	-	-.499* (.134)	-.510* (.134)
Medicare	-	-	reference	reference
Cash	-	-	-.419* (.138)	-.420* (.137)
Unknown payer	-	-	-.530* (.164)	-.530* (.164)
PC not visited	-	-	reference	reference
PC visited but not offered	-	-	.293* (.056)	.303* (.056)
PC visited and offered	-	-	-.289* (.058)	-.282* (.058)
Last test in the past 0-3mo	-	-	reference	reference
Last test in the past 4-6mo	-	-	.840* (.103)	.842* (.103)
Last test in the past 7-12mo	-	-	1.061* (.093)	1.063* (.093)
Last test in the past 13-24mo	-	-	1.373* (.099)	1.378* (.099)
Last test in the past 25-36mo	-	-	1.199* (.121)	1.208* (.121)
Last test in the past 37-60mo	-	-	1.482* (.128)	1.483* (.128)
Last test in the past 61mo+	-	-	1.139* (.094)	1.150* (.094)
Never been tested	-	-	.921* (.079)	.931* (.080)
Unknown last test	-	-	-.045 (.084)	-.040 (.084)
Metropolitan area	-	-	-	-.003 (.116)
Non-metropolitan area	-	-	-	reference
Population density per ZCTA	-	-	-	.020 (.028)
% below 200% poverty level per ZCTA^*c*^	-	-	-	.483* (.196)
Site #03: After protocol change	-	1.654* (.076)	1.655* (.078)	1.646* (.078)
Site #03: Before protocol change	-	reference	reference	reference
Site #01 (fewer than 50 patients)	-	-.283 (.343)	-.350 (.448)	-.238 (.439)
Site #02 (health fairs)	-	4.552* (.972)	4.321* (.983)	4.392* (.994)
Site #03	-	-.639* (.076)	-.924* (.087)	-.856* (.091)
Site #04	-	.496* (.099)	.491* (.101)	.399* (.097)
Site #05	-	.023 (.153)	.583 (.329)	.610 (.330)
Site #06 (fewer than 50 patients)	-	1.015* (.198)	.953* (.205)	1.012* (.202)
Site #07	-	.772* (.069)	.740* (.073)	.721* (.070)
Site #08	-	-2.257* (.085)	-1.728* (.096)	-1.748* (.095)
Site #09	-	.275* (.095)	.457* (.102)	.461* (.102)
Site #10	-	reference	reference	reference
Error variance (L2 intercept)	.231* (.033)	.027* (.011)	.017* (.009)	.006 (.008)
-2LL	17863.22	16001.55**	15101.40**	15081.02**

Note

*p < .05;

** = likelihood ratio test significant; ICC = .066; values based on SAS PROC GLIMMIX; entries show parameter estimates with standard errors in parentheses; estimation method = Laplace.

^a^ Best fitting model.

^b^ Including transgender women; none of the patients identified as a transgender man.

^c^ We tested different levels of poverty (from 50% to 500%) and found no noticeable difference in the results.

Model 4 shows that HIV screening experience at recent primary care visits is a significant determinant of screening test acceptance at dental visits. Those who have seen a primary care provider but have not been offered an HIV test are more likely to accept HIV screening at their dental visits than those who have not seen a primary care provider in the past 12 months. Also, those who have been offered HIV screening by a primary care provider are less likely to accept HIV screening at their dental visits.

Those who have never been tested and those who have been screened more than 3 months ago are significantly more likely to accept the screening offer than those who have been tested for HIV in the past 3 months.

Patient’s demographic characteristics are also significant predictors of screening acceptance. A patient is more likely to accept the HIV screening offer if the patient is younger (age 18–34), black/Hispanic/multi-racial (compared to white), born outside of the United States or Puerto Rico, male, or covered by Medicare, controlling for all other variables.

Of the three ZCTA-level variables, the poverty level is found significant: those who reside in economically disadvantaged communities are more likely to accept HIV screening.

### HIV screening from patient’s perspective

About 40% of the patients declined HIV screening. Among those who provided a reason of refusal, the most frequent reasons included: they had recently been screened for HIV (54%); they believed that they were not at risk for HIV (38%); or they reported other reasons (8%).

We also learned about patient’s concerns with HIV screening from conversations between clinical staff and patients. Confidentiality was identified as a major issue. Most patients preferred to have the HIV screening test administered with a degree of privacy. Due to the stigma of HIV, some patients wondered if their desire to be screened for HIV would implicate them in risk behaviors such as unprotected sex or intravenous drug use. Another major concern was the fear of positive results. Some patients declined the test offer because they were afraid to know their HIV status and worried that they could be discriminated against by others including the staff of the dental office if they were known to be HIV positive.

### HIV screening from provider’s perspective

Qualitative information on barriers, facilitators, and best practices were collected among clinical and non-clinical staff near the end of the project. Providers were mostly concerned over the practical issues of implementing HIV screening in the provision of routine dental care. Space and staff shortage were noted as barriers. Some sites did not have enough room for confidential testing on busy days with high patient volume. Some sites did not have enough staff to provide HIV screening, particularly when the trained dentists or hygienists were off work that day.

Other issues and concerns included: (1) salivary diagnostics and/or HIV screening might not be reimbursed in a dental office; (2) the accuracy of rapid HIV testing might vary and the psychological impact of false positive results could be significant; and (3) standardized training videos (in addition to the face-to-face training sessions) could be useful vehicles to train dentists/dental students and staff to embrace HIV testing in the dental setting.

Most providers reported that implementation of HIV screening into routine dental care was successful largely due to the following reasons: (1) The OraQuick™ tests were easy to administer; (2) results were obtained rapidly; and (3) patient acceptance was high enough to warrant screening. Faculty members of dental schools also acknowledged that the implementation of HIV screening in routine dental care was an effective educational tool for dental students to learn about HIV infection among asymptomatic individuals.

## Discussion and conclusion

Despite the need for expanded HIV screening amongst asymptomatic populations, and despite the continual discussion about HIV screening in dental setting, we find only four studies that have implemented HIV screening in dental care. To fill this gap, we implemented a project in NYS lasting 26 months to (1) assess the feasibility of HIV screening in dental setting, (2) identify the factors associated with patient acceptance of HIV screening, and (3) highlight best practices towards HIV screening in a dental setting. Our data indicate high test acceptance among dental patients and that a large percentage of dental patients have never been tested for HIV and many others were not screened for HIV by their primary care providers. Our findings suggest that implementing HIV screening as part of routine dental care to all dental patients regardless of individual risk factors can reduce stigma associated with HIV screening.

This study has several unique strengths. First, the sample size is much larger than those in similar studies in published literature. The sample included 14,935 patients who presented for dental care, of whom 9,057 (60.8%) were screened for HIV as part of routine care. In addition to providing a robust estimate of test acceptance, a large sample size enables statistical analyses of the effects of patients’ demographics, previous HIV testing experiences, and community characteristics on the likelihood of HIV screening acceptance. Second, this study covers multiple regions in NYS, a populous state in the United States. Dental patients were residing in 504 ZIP code areas, which accounted for 31% of all ZIP code areas in NYS. Third, our project is a multi-school public health initiative. HIV screening was implemented in multiple dental school clinics, community dental clinics, and private practices that served diverse populations across NYS.

Consistent with the findings reported by Pollack et al (2014),[15] we found that oral rapid HIV screening fit well into the provision of routine dental care. Dental providers and staff were able to administer the screening effectively after a short training period. Dental providers (dentists, dental students, and hygienists) who administered rapid HIV screening were able to link preliminary positive patients for confirmatory testing and subsequent medical care. Above all, dental providers demonstrated willingness to incorporate rapid HIV screening into routine practice. Furthermore, implementing HIV screening in routine dental care was an effective educational tool for dental students to learn about HIV infection among asymptomatic individuals.

We found that how the HIV screening test was offered could have potential impact on patient acceptance. First, offering an HIV screening test as part of routine dental care to all patients seemed to have contributed to a high test acceptance level by reducing perceived stigma. In practice, it was important to inform patients that all patients were offered HIV screening, regardless of risk factors. The availability and display of various promotional materials (such as posters and leaflets) were also useful tools for patient education prior to offering HIV screening to dental patients.

Furthermore, acceptance was higher if the offer was made by clinical staff than by administrative staff. This variation in acceptance rate resembled the findings of Nassry et al (2012), where the acceptance rates varied from 8% when the test offer came from administrative staff, to 88% when the test offer came from a faculty member or student.[29]

In addition to patient characteristics, factors associated with prior HIV testing experience were significant predictors of test acceptance. Patients were more likely to accept the screening offer if they had never been tested for HIV, or if they had not been tested for HIV recently. Furthermore, those who had seen a primary care provider in the past 12 months but had not been offered an HIV screening test were more likely to accept the screening offer at their dental appointment than those who had not seen a primary care provider in the past 12 months.

It is worth discussing the low seropositivity rate among the dental patients of this study. The 26-month project found one new confirmed HIV positive diagnosis among 9,057 tests performed, resulting in a 0.011% rate. This rate was higher than that of Bradley et al (2018)[27] and Nassry et al (2012),[29] which found no HIV positive persons, whereas this rate was lower than 0.31% in Suarez-Durall et al (2019),[26] 0.53% in Blackstock et al (2010)[28] or 0.13% in Leung et al (2016).[30] It is noteworthy that these studies found almost all of the HIV positive individuals in high-risk areas such as Los Angeles and New York City, and that the estimated percentage of people with undiagnosed HIV (among the adult population) was much higher in New York City (0.081%) than the rest of the state (0.037%) in 2017.[2] Thus, certain regional characteristics may have influenced the HIV positivity rates of the studies cited above. While routine HIV screening in dental setting is strongly encouraged for such areas as New York City, it is still encouraged for other areas, because much of the adult population is not screened for HIV by primary care providers even if primary care providers are required to offer an HIV screening test to their patients: 33% of dental patients had never been screened for HIV; and 56% of dental patients did not have an HIV screening opportunity with primary care providers in the past 12 months. Thus, dental clinics can effectively expand HIV screening efforts to individuals who are not frequently screened for HIV by primary care providers. The CDC also encourages routine HIV testing in all health care settings, as routine testing does not require a risk assessment, may help reduce stigma associated with HIV screening, and may increase patient acceptance of HIV screening.[3]

There are limitations to this study. First, in spite of the large sample size with broad regional coverage and the inclusion of community dental clinics and private practices, patients who presented for dental care at these clinic locations may be self-selected. Accordingly, study findings may not be generalizable to all dental patients in NYS. Second, HIV screening was offered at no cost to the patients. In addition, patients who accepted the screening tests did not have to be concerned about sensitive information being shared with their insurance companies. Patient acceptance of HIV screening might have been adversely impacted if patients were required to make a co-payment or pay out of pocket. Third, the study did not collect any information on risk factors that might be associated with HIV infection. The lack of risk factor data does not allow statistical control of self-reported risk factors in our test acceptance model.

We believe there are additional measures to be considered when assessing the implications of the findings and help inform future research. First, one-third of all dental patients included in the present study had never been tested for HIV. Among those who accepted the free test offer and got tested, 3,517 (39%) were first-time testers. In addition to being informed about their status, first-time testers were also provided with HIV prevention and treatment messages as appropriate. Second, among those who were offered an HIV screening test but refused, 1,444 (25%) remained never tested for HIV (i.e., unreached or hard-to-reach population). Third, our data also indicated that among those who refused the test offer, 1,502 (25%) had visited a primary care provider in the past 12 months but were not offered an HIV test during the visit. Of note, another 14% of those who refused, or 819 individuals, did not have primary care visit in the last 12 months. Two groups taken together, over 2,300 individuals, or 16% of all dental patients in our sample, may have experienced a *missed opportunity* of getting to know their HIV status within the healthcare system. Future studies may seek to develop new messaging, in terms of methods and contents, for the unreached and the *missed opportunity* populations.

Still further research seems beneficial on the cost-effectiveness analysis of routine HIV screening in dental setting. There are only a small number of published articles[34–40] on the cost-effectiveness of HIV screening in primary care setting. Our findings corroborate one of their suggestions: Routine HIV screening is more cost-effective in high-prevalence regions. One study further suggests routine HIV testing for not only patients but also those who accompany patients to the health care facilities in the communities of high HIV prevalence. [41] In general, our findings suggest that routine HIV screening in dental setting is beneficial where routine HIV screening in primary care setting is mandated, because our data show that a large percent of dental patients have never been tested for HIV, have not seen a primary care provider or have not been offered an HIV screening test by a primary care provider in the past year.

In addition, given the large number of studies of HIV screening in dental setting (mostly surveys of dental providers and patients), a systematic review or meta-analysis of them is also encouraged. While Silveira and Chattopadhyay’s paper[42] addresses many aspects of HIV screening in dental setting based on a review of many previously published studies, their paper is not based on a systematic review, needless to say that a number of studies have been published afterwards.
